# Risk attitudes and household consumption behavior: Evidence from China

**DOI:** 10.3389/fpubh.2022.922690

**Published:** 2022-09-06

**Authors:** Xin Xie, Zefeng Tong, Shulin Xu

**Affiliations:** ^1^School of Accounting, Guangzhou College of Commerce, Guangzhou, China; ^2^School of Public Finance and Administration, Zhongnan University of Economics and Law, Wuhan, China; ^3^School of Economics, Jinan University, Guangzhou, China

**Keywords:** risk attitude, household consumption, consumption behavior, consumption type, household finance survey, heterogeneity analysis

## Abstract

Risk attitude is a vital component of public mental health. Thus, the public should be guided to fully comprehend risks to improve public mental health. Using panel data from China Household Finance Survey (CHFS) in 2017, this study examined the impact of risk attitudes on household consumption behavior by constructing a micro-econometric model. Results suggest that risk attitude can promote household consumption, with multiple robustness tests supporting this conclusion. In addition, after dividing the consumption types into subsistence consumption, development consumption, and enjoyment consumption, we show risk preference promotes all three types of consumption and has the greatest impact on enjoyment consumption. Concurrently, risk neutrality can promote household survival consumption, but its promotion effect is smaller than that of risk preference. Moreover, risk aversion has an inhibitory effect on total consumption behavior, but this inhibitory effect does not show heterogeneity for different consumption behaviors. Heterogeneity analysis found that for male households, risk attitude remains an important factor in consumption behavior. When men's risk attitude is more risk averse, it can promote more survival consumption, whereas women's risk attitude is more risk averse. With increasing age, risk attitude remains a crucial factor in the occurrence of consumer behavior. However, education level has no bearing on the effect of risk attitude on household consumption behavior. This research holds theoretical and practical significance for improving public mental health, optimizing residents' consumption structure, and achieving high-quality economic development.

## Introduction

A growing body of literature suggests that risk attitudes determine individual behavior in multiple domains, such as saving and investment decisions (1, 2), consumption behavior (3), and career choice (4, 5). The risk attitude of residents is an important factor affecting the public's mental health. When the public maintains a diverse attitude toward risk, their mental health improves.

The rapid spread of COVID-19 has had a dramatic global impact on the mental health and lives of consumers, leading to major changes in public consumption concepts and consumption patterns (6). The pandemic has greatly increased people's demand for public health and food nutrition consumption. Therefore, changes in the consumption structure, especially the increase in consumption preferences in terms of nutrition acquisition and hygiene, are conducive to improving public health. Consumption is an indispensable factor in economic development (7). Consumer behaviors transfer the value of goods from producers to consumers. The policy of “expanding domestic demand” in China is to encourage consumption and investment. Stimulating consumption can lead to timely sales of goods, promote the normal operation of enterprises, and increase the employment rate. In such a consumption state, society can operate effectively and realize social reproduction. In addition, consumers' information on commodities is often lagging, and a serious information asymmetry exists between buyers and sellers (8). Consumers' purchasing decision behavior is a choice behavior under uncertainty and a risk-based decision. Therefore, a comprehensive, in-depth, and systematic study of consumer behavior can effectively promote consumption behavior.

In the face of the pandemic's impact and the complex international environment, the Chinese government has introduced a series of policies to stimulate market vitality and promote consumption recovery, including assisting corporate financing, accelerating the construction of short-term projects, promoting effective investment and industrial upgrading, and enhancing residents' social security. Under the influence of these policies, China's economy has achieved growth at a relatively fast rate, and domestic demand has recovered significantly (9). Meanwhile, the market sentiment has recovered, and residents' consumer confidence has rebounded strongly, which has laid a solid foundation for the accelerated high-quality development of the consumer market.

However, many studies on consumer behavior have not yet formed a complete theoretical system. As a result, research in this field remains in business investigation and discussion and lacks quantitative empirical analysis. In the era of the new economic normal, studying the impact of risk attitudes on residents' consumption behavior is not only of great social significance for understanding and guiding residents' consumption orientation and product market orientation, but also crucial to improve the consumption structure, alleviate employment pressure, promote social consumption, increase residents' income, and boost economic development.

Risk attitude is the mode of reaction that decision makers choose when faced with important uncertain outcomes. Generally, it can be divided into risk preference, risk neutrality, and risk aversion. Risk-averse people are more afraid and averse to risk occurrence than risk-neutral people and risk preference people, so they pay more attention to risk prevention (10, 11). When facing risks, risk neutrals show neither preference nor aversion but an unbiased attitude toward risks (12). Residents with risk preferences are more willing to take additional risks to obtain high returns (13).

Risk attitude is a stable trait that significantly influences decision-making behavior. Residents are always faced with consumption choices in their daily life, and their different risk attitudes lead to various decision-making behaviors. Rational households will make asset decisions in consumption, investment, and savings according to the economic environment to avoid risks and realize asset appreciation (14). Risk-averse investors always choose to invest in single or safe assets, whereas risk preference investors invest more in stocks and risk assets (15). Therefore, people with a risk preference will promote the diversification of household financial asset portfolios and achieve higher investment returns by optimizing household asset allocation (16). After households obtain a return on investment, they are more inclined to spend this portion of income on education, medical care, tourism, and other consumption, which increases household consumption behavior.

Residents' consumption behavior can be subdivided into subsistence consumption, development consumption, and enjoyment consumption. Subsistence consumption mainly refers to the consumption of food, clothing, and housing. Development consumption mainly refers to the consumption of education, transportation, and medical care. Enjoyment consumption mainly refers to household equipment, culture, and entertainment. Thus, will risk preference promote residents' consumption behavior? What kind of consumer behavior will it affect? To answer these questions, we conduct an empirical analysis of micro-survey data from the 2017 China Household Finance Survey (CHFS). On this basis, we also explore the impact of risk attitudes on household consumption behaviors of residents of different genders and ages to make targeted recommendations.

The possible contributions of this paper are as follows. Firstly, it enriches the literature on the impact of risk attitudes on residents' consumption behavior. Scholars have explained the influencing factors of residents' consumption behavior in the relevant literature, such as income, gender, and occupation (17, 18). However, studies related to household consumption behaviors on risk attitudes remain scant. Although some studies have focused on the impact of risk attitudes on residents' economic behavior, these studies mostly analyzed residents' investment and saving behaviors (2, 19, 20) and paid less attention to consumer behavior that is closely related to the daily life of residents. We discuss how risk attitudes affect residents' consumption behavior, which fills the gap in the existing literature. In addition, exploring the impact of risk attitudes on residents' consumption behavior enriches the literature on public mental health and individual decision making and provides new evidence on the impact of mental health on economic behavior.

Secondly, we not only analyse the impact of risk attitudes on household consumption in general, but also divide household consumption into subsistence consumption, development consumption, and enjoyment consumption and thoroughly analyse how risk preference, risk neutrality, and risk aversion affect different consumption patterns. Not only is this conducive to examining the impact of different risk attitudes on residents' consumption structure and consumption preferences, it also broadens the existing research on residents' consumption behavior.

Thirdly, we use comprehensive survey data to provide persuasive empirical support for the research. Most of the existing discussions on consumer behavior remain at the theoretical level and lack comprehensive empirical data support. We conduct empirical research with the help of the 2017 CHFS data. The survey data covers the micro-demographic data of 40,011 households in 29 provinces, 355 cities, and 1,428 villages in China, which provides a comprehensive and detailed data source.

Finally, we provide a more novel and comprehensive perspective for studying risk attitude and residents' consumption behavior. When studying the impact of risk attitudes on household consumption behavior, we consider the possible heterogeneity in many aspects. We constructed a model on the basis of the residents' gender, age, and educational level and added interaction terms to analyse the heterogeneity of the impact of risk attitudes on household consumption behaviors from the aspects of gender, age, and educational level. We provide a more novel and comprehensive perspective for related research.

The structure of this paper is as follows. The second section provides an overview of the recent literature on risk attitudes and household consumption behavior. The third section describes our research design, fourth section is the discussion of result, fifth section is the discussion of heterogeneity analysis, sixth section is the robustness check, and seventh section concludes the study and discusses the implications.

## Literature review

### Factors affecting risk attitudes

Studies on the influencing factors of risk attitudes have shown that different decision makers will present completely different risk attitudes under the influence of multi-faceted and multilevel factors. Factors affecting risk attitudes can be summarized as family, individual, and environmental factors.

#### Household factors

Family is an important factor affecting individual risk attitudes (21). Generally, the higher the income level of a family, the more net wealth it has accumulated, and thus the more ability to bear investment risks, the stronger the risk appetite. Sundar and Virmani (22) indicated that high- and low-income households are risk seeking and middle-income households are risk averse, and a U-shaped structure influences household income and risk attitudes. They also found that the larger the family size and the more educated the family members are, the more risk averse they are. Although the positive effect of high income on risk appetite has been confirmed, residents will show a more obvious risk aversion behavior (13). Fluctuations in wealth create risk aversion in households, which in turn affects their portfolio allocation (23). In addition, the more elderly and children in the family, the more it will reduce the risk appetite of the family and the investment in risky assets (16). Some studies have also analyzed the impact of marriage on individual risk attitudes. Halek and Eisenhauer (21) and Lin (24) revealed that married people are more risk averse. Dohmen et al. (1) made a similar point, and their analysis showed that widows are more risk averse than unmarried. Furthermore, Görlitz and Tamm (25) unveiled that being a parent makes individuals more risk averse.

#### Individual characteristics

Individual characteristics, including investors' gender, age, occupation, and other factors, will impact risk attitude. Grable (26) concluded that the gender, age, marital status, career choice, education level, financial literacy, and other characteristics of individual investors would impact investors' risk attitude and risk-taking level. Risk attitudes affect investors' ultimate achievement, which is largely influenced by personality traits and socioeconomic background. Influenced by previous investment experience, men have stronger financial risk-taking abilities than women and higher risk bias (27). Influenced by health conditions, older adults exhibit risk aversion and tend to choose safer investments than younger adults, but this risk aversion is attenuated in the presence of a partner (28). Dohmen et al. (29) studied how risk attitudes change over a person's lifespan and found that a person's risk appetite decreased throughout the lifespan, reaching a nadir at the age of 65, and then trending toward risk neutrality. In terms of occupational factors, freelancers are more willing to take financial risks and thus exhibit a higher risk appetite (30). In addition, from the cognitive ability perspective, Dohmen et al. (3) found that people lacking cognitive ability are more likely to become risk averse, and higher cognitive ability will increase the risk-taking willingness and ability of individuals, which in turn makes them become risk averse. Therefore, an individual's cognitive level also affects an individual's risk attitude, which in turn affects their economic decision making.

#### Environmental factors

The impact of environmental factors on risk attitudes includes family status, social changes, and other factors on risk attitudes. On the one hand, family environment and education have a subtle influence on adolescents' risk attitudes. Dohmen et al. (1) stated that parental education is important in shaping children's risk attitudes, and social mobility is intergenerational. On the other hand, changes in the socio-economic environment will change investors' risk attitudes. When the economic environment is unstable and residents face increased income uncertainty or restricted liquidity, they will become more risk averse (13). Rohde and Rohde (31) analyzed whether an individual's risk attitude is affected by the risk faced by others, and found that a person's behavior when faced with risk is affected by the risk attitude of others.

### Factors affecting household consumption behavior

Scholars have explored the influencing factors of residents' consumption behavior, which can be summarized as the influence of residents' characteristics, economic factors, and other factors.

#### Individual characteristics

Household consumption behavior will be affected by individual characteristics, such as gender, age, education level, and other factors. The literature on consumer behavior reported significant differences in emotional and cognitive processes between men and women, leading to differences in consumer behavior decision making between men and women (32). Compared with men, women are more likely to engage in hedonic consumption and impulse purchase (33). In terms of age, Guiso et al. (34) pointed out a non-linear relationship between age and asset selection in the study of household financial asset selection. The relationship between age and the allocation ratio of financial risk assets is an inverted U shape, which confirms that the allocation of household financial risk assets has obvious life cycle effects. However, the higher the education level of the household head, the more likely the household is to participate in stock market investments (35). Staddon et al. (36) claim that individual psychological conditions such as attitudes also influence consumption behavior. As educational attainment increases, households engage in more exploratory consumption behaviors (37). In addition, the persistence of residents' consumption behavior is affected by key factors, such as personal values, attitudes, and income (38).

#### Economic factors

Economic factors will influence the characteristics of consumer behavior (39). With the development of the internet economy and the maturity of digital technology, the popularization of digital finance has promoted the consumption of residents (40). In the consumption category, digital amounts show a clear positive correlation between finance and food, clothing, home maintenance, medical care, and education and entertainment. Amongst households with fewer assets, lower income, and lower financial literacy and households in small- and medium-sized cities, digital inclusion has a greater effect on consumption (41). Although residents' income and consumption habits will affect residents' consumption behavior, food, cash, housing, and energy will remain the key consumption areas (42). In the context of the development of a circular economy, residents have begun to pay increasing attention to green consumption under the influence of consumer values and behavior habits. The concept of green consumption will affect the daily behavior of residents in catering, work, and transportation, thereby promoting the development of a more environment-friendly, sustainable consumption, and production system (43). In addition, some studies also analyze the impact of economic factors such as household debt risk (44), household liquidity constraints (45), and household financial vulnerability (46) on household consumption behavior.

#### Other factors

In addition to the influence of individual characteristics and economic factors, other factors will impact household consumption behavior. Wang et al. (47) found that urban residents' daily energy consumption behavior is mostly driven by “altruism,” and social norms and policy environment greatly impact residents' energy consumption behavior. Zhang et al. (48) proposed that residents' perception of haze pollution (HPP) will enhance residents' willingness to consume green products and promote green consumption behavior. Moreover, Alexander and Karger (49) studied the impact of the new crown epidemic on consumption and concluded that the outbreak of the COVID-19 would restrict residents' travel, thereby reducing residents' consumption in the mobility industry. In addition, some scholars have also studied the impact on household consumption behavior from the aspects of family lifestyle (50), gender inequality (51), age structure (52), and financial development (51).

### Factors affecting residents' financial behavior decisions

Individual risk and family risk attitudes will impact residents' financial decision-making behavior. Individual investors' risk preference for potential benefits and losses shows significant differences. Risk-averse investors have a less total investment in financial assets, whereas risk-averse investors behave in the opposite way. The degree of participation of family members and family assets will affect the family's risk attitude, which will impact family financial decision making and investment portfolio behavior (53).

#### Individual risk attitude

Risk attitudes play a fundamental role in individual decision making (54). A growing body of research shows that individuals' attitudes toward risk largely influence their decisions on consumption, financial investment, and employment (1, 2, 55). Individual decision makers' varying degrees of risk preference will lead to the different distribution proportions of risk assets and risk-free assets. Gollier (56) proposed that the different degree of residents' risk preference will change their financial asset portfolio. Risk-averse investors tend to prefer holding portfolios dominated by safe assets, whereas risk-averse investors' portfolios are mainly risky assets, and moderately risk-averse investors tend to hold the most diversified portfolios (15). Scholars have confirmed the relationship between individual investment risk attitudes and the portfolio of financial assets held by household survey data, such as Barasinska et al. (19). They have confirmed that risk averters tend to hold portfolios mainly comprising risk-free assets by using German household survey data. Conversely, risk preference is more inclined to hold riskier financial assets. Investors invest in risky financial assets, and this behavior significantly impacts the increase in returns (57). The higher the tolerance of risk, the more equity investment assets owned by individual investors, which contributes to the wealth accumulation of residents (58). In addition, changes in health status impact residents' financial portfolios. When a person's health is negatively affected, their wealth plummets, thus influencing their investment decisions in the future (59).

#### Family risk attitude

Serra-Garcia (54) argued that, in addition to individuals' attitudes toward risk, the composition of households' risk attitudes impacts residents' behavior. Most family decisions are made jointly, and parents can influence family decision-making behavior. In joint family decision making, the husband usually has a greater impact on family decision making than the wife (60). Many existing studies have analyzed the impact of household risk on financial behavior decisions. The life cycle permanent income theory proposed by Ando et al. (61) holds that households will achieve intertemporal optimization of resource allocation through balanced allocation of financial assets and real assets, such as real estate, thereby smoothing consumption expenditures. When husband and wife have different risk preferences, the family's investment in risky financial assets in financial asset allocation generally depends on the partner with higher education and risk tolerance (62). In the family, women are generally more risk averse than men, but when the wife is more risk tolerant than her husband or the wife has less financial experience, men's risk preference has a greater impact on family joint decision making (27). An increase in a household's property value or mortgage debt reduces the household's risk preference, which reduces the household's propensity to participate in the stock market and the equity share of household assets (63). Amongst risk-oriented households, an adjustable rate (ARM) mortgage is often chosen over a fixed rate (FRM) mortgage (64). In addition, households with different risk attitudes have obvious heterogeneity in consumption. Zhang et al. (65) studied the impact of stock market crash on household consumption and found that risk-averse and risk-neutral households reduced consumption after the stock market crash, but risk-averse households chose to minimize consumption. A study by Barasinska et al. (19) using data from the German Household Survey found that risk-averse households tend to hold a portfolio consisting mainly of risk-free assets.

Based on the above literature, most scholars recognize the influencing factors of risk attitude and household consumption behavior and study the impact of risk attitude on household financial decision-making behavior, which can provide a reference for our study. However, scholars have less special analysis on how risk attitude affects residents' consumption behavior and what kind of consumer behavior it affects. Therefore, the latest data can be used to conduct an empirical analysis of the impact of risk attitudes and household consumption behaviors. With the help of the 2017 CHFS data, we established a model to conduct empirical research and in-depth discussions on risk attitudes and household consumption behaviors.

## Research design

### Data

Our data is from a survey conducted by the China Household Finance Research Center of Southwestern University of Finance and Economics. The survey covers micro-demographic data of 40,011 households in 29 provinces, 355 cities, and 1,428 villages in China, which provides a more comprehensive and scientific data source for this paper to study the impact of risk attitudes on household consumption behavior.

### Models and variables

Residents' risk attitude and consumption behavior were modeled as follows. In the model, the explained variable is the total consumption, defined as TC and the explanatory variable is risk attitude, defined as Risk. Many related variables are also controlled, which reflect the individual characteristics of residents and family characteristics, such as gender, age, education, marital status, total family assets, and income. These control variables are collectively called variable *Z*, and *e* is a random disturbance term.


(1) $$\begin{array}{l} TC=\alpha_{0}+\alpha_{1}\times Risk+\alpha_{3}\times Z+\varepsilon_{i} \end{array}$$

On the basis of the different types of household consumption behavior, the variable *TC* was subdivided into subsistence consumption (*SC*), development consumption (*DC*), and enjoyment consumption (*EC*) and we construct Models (2)–(4). The answers to several questions about consumption expenditure in the CHFS were counted, and the total household consumption annual amount and the subsistence consumption annual amount (including food, clothing, housing, and so on), development consumption annual amount (including education, transportation, medical treatment, and so on), and enjoyment consumption annual amount (including household equipment, culture, and entertainment and so on) were calculated. In addition, the amounts of *TC*, *SC*, *DC*, and *EC* are processed logarithmically in our model.


(2) $$\begin{array}{l} SC=\beta_{0}+\beta_{1}\times Risk+\beta_{3}\times Z+\varepsilon_{i} \end{array}$$

(3) $$\begin{array}{l} DC=\gamma_{0}+\gamma_{1}\times Risk+\gamma_{3}\times Z+\varepsilon_{i} \end{array}$$

(4) $$\begin{array}{l} EC=\delta_{0}+\delta_{1}\times Risk+\delta_{3}\times Z+\varepsilon_{i} \end{array}$$

Risk attitude is an explanatory variable that has three types: risk preference, risk neutrality, and risk aversion. Risk attitude indicators were constructed according to the different options of respondents' questions about investment risk attitudes in the questionnaire. Respondents who choose Options 1 and 2 of the questionnaire were regarded as risk preference; respondents who choose Option 3 as risk neutrality; the respondents who choose Options 4 and 5 as risk aversion. The data of Option 6 were excluded from the sample. Amongst them, risk preference was assigned 1, risk neutral was assigned 2, and risk aversion was assigned 3, which constitutes a comprehensive index of risk attitude.

Referring to the practice of previous literature, multivariable control was carried out, which can be divided into two categories. The first category is personal characteristic variables, including gender, age, education level, marital status, health status, political identity, and business background.

The second type of control variable is family characteristic variables, including the number of houses, total family assets and family income. The specific definitions of variables are shown in Table 1.

**Table 1:** The specific definitions of variables.

**Type**	**Symbol**	**Definition**	**Explanation**
Explained variables	TC	Total household consumption	Natural logarithm of total consumption
	SC	Subsistence consumption	Natural logarithm of subsistence consumption
	DC	Developmental consumption	Natural logarithm of developmental consumption
	EC	Enjoyment consumption	Natural logarithm of enjoyment consumption
Explanatory variables	Risk	Risk attitude	Risk aversion is 1, risk neutrality is 2, and risk preference is 3
	Risk-P	Risk preference	Risk preference are 1, others are 0
	Risk-N	Risk neutrality	Risk neutrality are 1, others are 0
	Risk-A	Risk aversion	Risk aversion are 1, others are 0
Control variables (Z)	GE	Gender	1 for males, 0 for females
	AG	Age	Actual age of head of household
	AG2	Age2	The square of the actual age of the head of the household
	EL	Education level	The education level of the head of household is 1–9 for primary school and below, primary school, junior high school, technical secondary school, high school, junior college, undergraduate, master, and doctoral degree
	MS	Marital status	1 for married or cohabiting, 0 for others
	HS	Health status	1 if the head of household is healthy, 0 otherwise
	PI	Political identity	1 if you are a member of the Communist Party of China or a probationary member, otherwise 0
	BB	Business background	1 if engaged in industrial and commercial operation, otherwise 0
	NH	Number of houses	The number of houses owned by the family
	FA	Total family assets	Natural logarithm of total family assets
	FI	Family income	Natural logarithm of total family income

## Results

### Descriptive statistics

The descriptive statistics of the variables are exhibited in Table 2. The mean of risk attitude in the sample is 1.514, indicating that the risk attitude of most residents in China tends to be neutral as a whole. The mean of education level is 3.430, which explains that the respondents were mostly secondary educated. Respondents were mostly middle aged, with an average age of 55, and the oldest is 117. The means of respondents' gender, marital status, health status, and political identity are 0.793, 0.850, 0.476, and 0.126, respectively. These results indicate that the household heads in the sample are predominantly male and mostly married. In addition, most of the respondents are healthy and not members of the Communist Party of China. The mean of the respondents' business background is 0.143, indicating that most residents did not engage in industrial and commercial operations. Ultimately, most families own their own homes, and the average number of homes owned by a family is 1.221.

**Table 2:** Descriptive statistics results.

**Variables**	**Observations**	**Mean**	**SD**	**Median**	**Min**	**Max**
TC	40,011	10.62	0.870	10.67	6.604	13.82
SC	40,001	10.57	0.958	10.65	5.889	15.15
DC	40,010	9.923	2.002	9.904	0	16.74
EC	39,016	5.371	4.666	6.399	0	16.30
Risk	10,681	1.514	0.718	1	1	3
Risk-P	13,193	0.107	0.310	0	0	1
Risk-N	13,193	0.202	0.401	0	0	1
Risk-A	13,189	0.501	0.500	1	0	1
GE	40,010	0.793	0.405	1	0	1
AG	40,000	55.20	14.25	55	17	117
AG2	40000	3250	1589	3025	289	13689
EL	39,958	3.430	1.684	3	1	9
MS	39,966	0.850	0.357	1	0	1
HS	40,002	0.476	0.499	0	0	1
PI	36,258	0.126	0.332	0	0	1
BB	40,010	0.143	0.350	0	0	1
NH	36,163	1.221	0.538	1	0	27
FA	40,011	12.56	2.005	12.85	0	17.22
FI	39,445	10.55	1.739	10.91	0	15.43

Data is from: China Household Finance Questionnaire (2017) of Southwestern University of Finance and Economics. The total household assets and household income are both abbreviated by 0.01 and logarithmic.

The descriptive statistics of household consumption behavior revealed that only the minimum value of subsistence consumption in the sample is not 0, which shows the subsistence consumption behavior is necessary for everyone. In addition, the means of developmental consumption and enjoyment consumption are 9.923 and 5.371, respectively, and the standard deviations are 2.002 and 4.666 (higher than the standard deviation of subsistence consumption of 0.958), indicating that the amount of these two types of consumption behaviors varies greatly amongst different families, especially for enjoyment consumption.

### Empirical results

#### Impact of risk attitude on household consumption behavior

As shown in Table 3, the OLS models were designed with five groups of different variables to report the estimated results of the impact of risk attitudes on household consumption behavior. Model (1) only includes risk attitude and total consumption variables. Model (2) adds control variables for individual residents and their family characteristics on the basis of Model (1). Model (3) is the model of risk preference and total consumption and controls other control variables. Similar to Model (3), Models (4) and (5) change the variables of risk preference into risk neutrality and risk aversion to explore the impact of different risk types on total consumption.

**Table 3:** The influence of risk attitude on household consumption behavior.

	**(1)**	**(2)**	**(3)**	**(4)**	**(5)**
	**TC**	**TC**	**TC**	**TC**	**TC**
Risk	0.252*** (0.010)	0.066*** (0.011)			
Risk-P			0.089*** (0.023)		
Risk-N				0.079*** (0.016)	
Risk-A					−0.035*** (0.013)
GE		−0.072*** (0.017)	−0.086*** (0.016)	−0.084*** (0.016)	−0.085*** (0.016)
AG		−0.016*** (0.003)	−0.016*** (0.003)	−0.016*** (0.003)	−0.016*** (0.003)
AG2		0.000**	0.000**	0.000**	0.000**
		(0.000)	(0.000)	(0.000)	(0.000)
EL		0.056*** (0.005)	0.066*** (0.005)	0.066*** (0.005)	0.067*** (0.005)
MS		0.288*** (0.024)	0.285*** (0.022)	0.283*** (0.022)	0.284*** (0.022)
HS		−0.035** (0.014)	−0.042*** (0.013)	−0.042*** (0.013)	−0.042*** (0.013)
PI		0.036** (0.018)	0.050*** (0.017)	0.049*** (0.017)	0.050*** (0.017)
BB		0.191*** (0.021)	0.209*** (0.020)	0.208*** (0.020)	0.209*** (0.020)
NH		0.088*** (0.015)	0.091*** (0.015)	0.093*** (0.014)	0.092*** (0.015)
FA		0.148*** (0.007)	0.144*** (0.005)	0.144*** (0.005)	0.146^**^* (0.005)
FI		0.102*** (0.007)	0.095*** (0.005)	0.095*** (0.005)	0.095*** (0.005)
Constant	10.472***	7.764***	7.957***	7.951***	7.957***
	(0.018)	(0.117)	(0.098)	(0.098)	(0.099)
*N*	10,681	8,009	9,956	9,956	9,954
*F*-value	575.673***	365.550***	512.483***	517.461***	511.717***
*R* ^2^	0.051	0.422	0.439	0.439	0.438

*** and ** indicate significance at the 1% and 5% confidence levels, respectively, with standard errors in brackets and coefficients outside brackets.

The results of Model (1) exhibit that when only considering the risk attitude variables, the coefficient of risk attitude is 0.252, which is significantly positive at the 1% level, indicating that the risk attitude has a significant positive impact on residents' household consumption and will positively impact residents' household consumption behavior. Model (2) demonstrates that the coefficient of risk attitude decreases from 0.252 in Model (1) to 0.066 in Model (2) when other variables were controlled, which is also significantly positive at the 1% level. The *P*-values of other control variables are all significant, showing strong robustness, further indicating that risk attitudes will impact household consumption. Under the condition that other control variables remain unchanged, the probability of household consumption behavior ratio will increase significantly by 0.066 (mean is 10.62) for each additional unit of risk attitude.

The explanatory variables in Models (3)–(5) are risk preference, risk neutrality and risk aversion, respectively. The coefficient of risk preference in Model (3) is 0.089, and it is significantly positive at the 1% level, indicating that risk preference will significantly promote household consumption under the control of relevant variables. The coefficient of risk neutrality of Model (4) is also significantly positive, showing that risk neutrality will positively impact household consumption behavior, which is similar to risk attitude. However, the coefficient is 0.079 in Model (4) (<0.089), indicating that each additional unit of risk preference can promote household consumption more than risk neutrality. The results of Model (5) show that the coefficient of risk aversion is −0.035, which is significantly negative at the 1% level, indicating that risk aversion has a significant negative impact on household consumption. The possible reason is that risk-averse people are more inclined to save money to avoid risks, thus inhibiting household consumption behavior.

On the basis of the results of Models (2)–(5), the relationship between control variables and explained variables can be analyzed. Firstly, males consume less than females, and the older the household head is, the lower the total consumption amount. With the improvement of the education level of the household head, the amount of various consumption behaviors will increase. Married people spend more than their unmarried counterparts due to higher household spending and childrearing. People with better health spend less, possibly because they spent less on things, such as medical care. Political identity is a member of the Communist Party of China, or the family who has been engaged in industrial and commercial operation has more consumption expenditure. In addition, the more assets, income, and houses a family owns, the higher the amount of consumption of the family.

#### Impact of risk attitude on different consumption types of households

The above analysis reveals that risk attitude will impact household consumption behavior, but how will various risk attitudes affect different types of household consumption behavior (survival consumption, development consumption, enjoyment consumption) remains unresolved. To address this problem, the explanatory variables were changed from total consumption to survival consumption, development consumption, and enjoyment consumption, and other control variables remain unchanged. Three groups of OLS models were designed for empirical research. Tables 4–6 report the estimated results of the impact of risk preference, risk neutrality, and risk aversion on different consumption types of residents' families. Columns (1)–(3) in the table are the results of the impact of risk attitudes on household subsistence consumption, developmental consumption, and enjoyment consumption.

**Table 4:** The impact of risk preference on different consumption types of households.

	**(1)**	**(2)**	**(3)**
	**Subsistence consumption**	**Developmental consumption**	**Enjoyment consumption**
Risk-P	0.103*** (0.024)	0.151** (0.061)	0.666*** (0.131)
GE	−0.084*** (0.016)	−0.176*** (0.047)	−0.602*** (0.098)
AG	−0.005* (0.003)	−0.020** (0.009)	−0.091*** (0.018)
AG2	−0.000***	−0.000	0.001***
	(0.000)	(0.000)	(0.000)
EL	0.065*** (0.005)	0.097*** (0.013)	0.659*** (0.029)
MS	0.252*** (0.021)	0.778*** (0.064)	0.488*** (0.124)
HS	0.055*** (0.014)	−0.351*** (0.039)	0.048 (0.086)
PI	0.066*** (0.017)	0.163*** (0.048)	0.457*** (0.105)
BB	0.169*** (0.021)	0.359*** (0.055)	0.192 (0.122)
NH	0.074*** (0.015)	0.212*** (0.038)	0.373*** (0.080)
FA	0.156*** (0.005)	0.151*** (0.014)	0.548*** (0.028)
FI	0.110*** (0.006)	0.152*** (0.013)	0.332*** (0.029)
Constant	7.475*** (0.105)	6.635*** (0.290)	−4.706*** (0.591)
*N*	9,955	9,956	9,681
*F*-value	643.261***	176.044***	503.521***
*R* ^2^	0.491	0.206	0.310

***, ^**^, and ^*^ indicate significance at the 1%, 5%, and 10% confidence levels, respectively, with standard errors in brackets and coefficients outside brackets.

**Table 5:** The impact of risk neutrality on different consumption types of households.

	**(1)**	**(2)**	**(3)**
	**Subsistence consumption**	**Developmental consumption**	**Enjoyment consumption**
Risk-N	0.075*** (0.017)	0.140*** (0.048)	0.651*** (0.101)
GE	−0.081*** (0.016)	−0.172*** (0.047)	−0.589*** (0.098)
AG	−0.005* (0.003)	−0.020** (0.009)	−0.089*** (0.018)
AG2	−0.000*** (0.000)	−0.000 (0.000)	0.001*** (0.000)
EL	0.065*** (0.005)	0.096*** (0.013)	0.656*** (0.029)
MS	0.250*** (0.021)	0.774*** (0.065)	0.473*** (0.124)
HS	0.055*** (0.014)	−0.351*** (0.039)	0.047 (0.086)
PI	0.066*** (0.017)	0.163*** (0.048)	0.455*** (0.104)
BB	0.169*** (0.021)	0.358*** (0.055)	0.186 (0.122)
NH	0.076*** (0.015)	0.215*** (0.038)	0.383*** (0.080)
FA	0.156*** (0.005)	0.151*** (0.014)	0.546*** (0.028)
FI	0.110*** (0.006)	0.152*** (0.013)	0.329*** (0.029)
Constant	7.473*** (0.106)	6.623*** (0.290)	−4.770*** (0.592)
*N*	9,955	9,956	9,681
*F*-value	649.308***	176.810***	510.918***
*R* ^2^	0.491	0.206	0.311

***, **, and * indicate significance at the 1%, 5%, and 10% confidence levels, respectively, with standard errors in brackets and coefficients outside brackets.

**Table 6:** The impact of risk aversion on different consumption types of households.

	**(1)**	**(2)**	**(3)**
	**Subsistence consumption**	**Developmental consumption**	**Enjoyment consumption**
Risk-A	−0.009 (0.014)	−0.020 (0.037)	−0.084 (0.082)
GE	−0.082*** (0.016)	−0.174*** (0.047)	−0.595*** (0.098)
AG	−0.006* (0.003)	−0.021** (0.009)	−0.094*** (0.018)
AG2	−0.000*** (0.000)	−0.000 (0.000)	0.001*** (0.000)
EL	0.066*** (0.005)	0.099*** (0.013)	0.667*** (0.029)
MS	0.251*** (0.021)	0.775*** (0.065)	0.479*** (0.124)
HS	0.055*** (0.014)	−0.351*** (0.039)	0.050 (0.086)
PI	0.067*** (0.017)	0.163*** (0.048)	0.457*** (0.105)
BB	0.170*** (0.021)	0.360*** (0.055)	0.197 (0.122)
NH	0.076*** (0.015)	0.214*** (0.038)	0.383*** (0.080)
FA	0.157*** (0.005)	0.153*** (0.014)	0.554*** (0.028)
FI	0.111*** (0.006)	0.153*** (0.013)	0.334*** (0.029)
Constant	7.493*** (0.106)	6.656*** (0.291)	−4.612*** (0.595)
*N*	9,953	9,954	9,680
*F*-value	640.094***	174.886***	499.913***
*R* ^2^	0.490	0.205	0.308

***, **, and ^*^ indicate significance at the 1%, 5%, and 10% confidence levels, respectively, with standard errors in brackets and coefficients outside brackets.

#### Impact of risk preference on different consumption types of households

Table 4 shows the impact of risk preference on different consumption types of households. Firstly, the results of subsistence consumption in Column (1) demonstrate that the coefficient of risk preference is 0.103, which is significantly positive at the 1% level, and the *P*-values of other variables are significant, indicating that risk preference significantly promotes the survival consumption of residents' families. Secondly, the regression results of risk preference on household development consumption are shown in Column (2) of the table. The coefficient of risk preference is 0.151, which is significantly positive at the 5% level, indicating that risk preference can also have a significant positive impact on residents' family development consumption behavior. Thirdly, the results in Column (3) of the table exhibit that the regression coefficient of risk preference on household enjoyment consumption is significantly positive at the 1% level, indicating that risk preference also plays a positive role in promoting enjoyment consumption. The coefficient is 0.666, which is higher than the coefficients of survival consumption and development consumption, indicating that risk preference has the most obvious promoting effect on enjoyment consumption. Overall, risk preference has a promoting effect on three types of consumption, and enjoyment consumption is most affected by risk attitude.

#### Impact of risk neutrality on different consumption types of households

Table 5 shows the regression results of the impact of risk neutrality on various household consumption. From the results of survival consumption in Column (1), the coefficient of risk neutrality is 0.075, which is significantly positive at the 1% level, indicating that risk neutrality can promote survival consumption of residents' families. Compared with the results of risk preference, the coefficient of risk neutrality on survival consumption is smaller than that of risk preference (0.103), indicating that the promotion effect of risk neutrality on survival consumption is smaller than that of risk preference. In Columns (2) and (3), the coefficients of risk neutrality are 0.140 and 0.651, respectively, and both of which are significantly positive at the 1% level and are similar to the corresponding coefficients of risk preference, showing that risk neutrality will also significantly positively affect the developmental consumption and enjoyment consumption.

#### Impact of risk aversion on different consumption types of households

The effect of risk aversion on different consumption types of households is shown in Table 6. The testing unveiled that the influence coefficients of risk aversion on household consumption for survival, development, and enjoyment were −0.009, −0.020, and −0.084, respectively, but they were not statistically significant, so it cannot be proved that risk aversion can affect which type of consumption is affected. Conclusively, although risk aversion have an inhibitory effect on the total consumption behavior, this inhibitory effect is not particularly obvious for various consumption behaviors. This may be because risk aversion is more about limiting the total consumption amount, and there exists no obvious tendency toward various consumption behaviors.

To sum up, different types of risk attitudes affect different consumption behaviors of residents' families distinctly. Risk preference and risk neutrality will positively impact different types of consumption behaviors of residents' families, whereas risk aversion does not significantly impact different types of consumption behaviors of residents' families. Amongst them, the positive impact of risk preference on household enjoyment consumption is greater than its positive impact on survival consumption and development consumption. The positive impact of risk preference on household subsistence consumption is higher than the positive impact of risk neutrality on survival consumption.

## Heterogeneity analysis

The literature review section indicates that individual characteristics (such as gender, age and education level and so on) affect residents' consumption behavior variedly (32, 33, 37), considering that the impact of risk attitude on household consumption behavior may be heterogeneous, such as gender, age, and education level. Therefore, the heterogeneity of these factors was analyzed, and the differences in household consumption behaviors of residents under different characteristics of risk attitudes were explored. In the field of econometrics, the interaction term is the product of two or more explained variables, which can indicate whether the influence of the core explained variable on the explanatory variable is related to other explained variables (adjustment variables). Therefore, next, the interaction item was constructed on the basis of the original model, the risk attitude was taken as the core explanatory variable of the interaction item and the gender, age, and education level of the head of household were selected as the regulatory variables of the interaction items. The following models are constructed.


(5) $$\begin{array}{l} TC=\alpha_{0}^{\prime }+\alpha_{1}^{\prime }\times Risk+\alpha_{2}^{\prime }\times Risk\times Trait+\alpha_{3}^{\prime }\times Z+\varepsilon_{i} \end{array}$$

(6) $$\begin{array}{l} SC=\beta_{0}^{\prime }+\beta_{1}^{\prime }\times Risk+\beta_{2}^{\prime }\times Risk\times Trait+\beta_{3}^{\prime }\times Z+\varepsilon_{i}^{\prime } \end{array}$$

(7) $$\begin{array}{l} DC=\gamma_{0}^{\prime }+\gamma_{1}^{\prime }\times Risk+\gamma_{2}^{\prime }\times Risk\times Trait+\gamma_{3}^{\prime }\times Z+\varepsilon_{i}^{\prime } \end{array}$$

(8) $$\begin{array}{l} EC=\delta_{0}^{\prime }+\delta_{1}^{\prime }\times Risk+\delta_{2}^{\prime }\times Risk\times Trait+\delta_{3}^{\prime }\times Z+\varepsilon_{i}^{\prime } \end{array}$$

Trait variables in the model represent the moderating variables of individual characteristics, including gender, age, and education level. The risk attitude was assigned a value of 1–3, which is a continuous core explanatory variable. The larger the value, the higher the risk preference of residents. Gender was assigned a value of 0 (female) or 1 (male), which is a dummy moderator variable. The assignment of age is the actual age of the head of household in 2017, which is also a continuous moderating variable. The greater the assignment, the greater the age. Education levels are assigned a value of 1–9 (although education levels are discrete, they do not matter), with higher values indicating higher education levels for the head of the household. The interaction between risk attitude and these moderator variables can reflect the heterogeneity of the impact of risk attitude on household consumption behavior.

### Gender heterogeneity

After introducing the interaction term of risk attitude and gender (*Risk*×*GE*) for the OLS regression, the estimated regression results are shown in Table 7. The results reveal that except for the regression coefficient of the interaction item in survival consumption, which is significant at the 10% level, the coefficients of the other interaction items are not significant. The coefficient of the interaction term in survival consumption is −0.049 and is significantly negative, which statistically proves the existence of the interaction effect between risk attitude and gender. Thus, compared with female residents, the negative impact of male residents' risk attitude on survival consumption is more significant. Therefore, gender differences exist in the impact of risk attitudes on household consumption behavior, and a moderating effect exists between risk attitudes and gender. For male-headed households, risk attitude remains an important factor in consumer behavior. When men's risk attitude tends toward risk aversion, it can promote more survival consumption, whereas women's risk attitude has a lower impact on residents' family consumption behavior than men, which reflects the typical gender heterogeneity of the impact of risk attitude on residents' family consumption behavior.

**Table 7:** Results of gender heterogeneity analysis.

	**(1)**	**(2)**	**(3)**	**(4)**
	**Total consumption**	**Subsistence consumption**	**Developmental consumption**	**Enjoyment consumption**
Risk	0.078*** (0.022)	0.103*** (0.024)	0.105* (0.062)	0.512*** (0.126)
Risk × GE	−0.016 (0.025)	−0.049* (0.026)	−0.005 (0.069)	−0.100 (0.141)
GE	−0.049 (0.040)	−0.006 (0.042)	−0.193* (0.115)	−0.425* (0.247)
AG	−0.016*** (0.003)	−0.004 (0.003)	−0.012 (0.010)	−0.090*** (0.020)
AG2	0.000** (0.000)	−0.000*** (0.000)	−0.000 (0.000)	0.001*** (0.000)
EL	0.056*** (0.005)	0.055*** (0.005)	0.093*** (0.014)	0.595*** (0.031)
MS	0.287*** (0.024)	0.254*** (0.024)	0.787*** (0.073)	0.506*** (0.143)
HS	−0.035** (0.014)	0.055*** (0.015)	−0.303*** (0.043)	0.048 (0.095)
PI	0.036** (0.018)	0.051*** (0.019)	0.132*** (0.051)	0.430*** (0.113)
BB	0.191*** (0.021)	0.150*** (0.022)	0.340*** (0.058)	0.008 (0.130)
NH	0.088*** (0.015)	0.070*** (0.016)	0.199*** (0.040)	0.349*** (0.083)
FA	0.148*** (0.007)	0.159*** (0.006)	0.144*** (0.016)	0.590*** (0.034)
FI	0.102*** (0.007)	0.116*** (0.007)	0.159*** (0.015)	0.351*** (0.035)
Constant	7.744*** (0.121)	7.262*** (0.124)	6.339*** (0.338)	−5.843*** (0.698)
*N*	8,009	8,008	8,009	7,797
*F*-value	337.452***	424.194***	119.018***	340.801***
*R* ^2^	0.422	0.466	0.189	0.294

***, ^**^, and ^*^ indicate significance at the 1%, 5%, and 10% confidence levels, respectively, with standard errors in brackets and coefficients outside brackets.

### Age heterogeneity

After introducing the interaction term of risk attitude and age (*Risk*×*AG*) for the OLS regression, the estimated regression results are shown in Table 8. The results demonstrate that the risk attitude is affected by age, the coefficient of the interaction term is only significant in the results of developmental consumption, and the coefficients of the other interaction terms are not significant. The coefficient of the interaction term in developmental consumption is 0.004, and is significantly positive at the 10% level, which proves the existence of the interaction effect between risk attitude and age. With the increase of age, the promotion effect of risk attitude on developmental consumption is more obvious, which may be due to the higher expenditure and demand for education and medical care as the age increases. Therefore, age differences exist in the impact of risk attitude on household consumption behavior, and a linkage effect exists between risk attitude and age. With the increase of age, risk attitude remains an important factor in consumer behavior. When risk attitude tends toward risk preference, it can promote more developmental consumption, which also reflects the typical age heterogeneity of the impact of risk attitude on household consumption behavior.

**Table 8:** Results of age heterogeneity analysis.

	**(1)**	**(2)**	**(3)**	**(4)**
	**Total consumption**	**Subsistence consumption**	**Developmental consumption**	**Enjoyment consumption**
Risk	0.112*** (0.040)	0.114*** (0.042)	−0.095 (0.111)	0.527** (0.231)
Risk × AG	−0.001 (0.001)	−0.001 (0.001)	0.004* (0.002)	−0.002 (0.005)
GE	−0.072*** (0.017)	−0.079*** (0.018)	−0.199*** (0.050)	−0.573*** (0.107)
AG	−0.013*** (0.004)	−0.002 (0.004)	−0.024** (0.012)	−0.085*** (0.025)
AG2	0.000* (0.000)	−0.000*** (0.000)	−0.000 (0.000)	0.001*** (0.000)
EL	0.055*** (0.005)	0.055*** (0.005)	0.094*** (0.014)	0.595*** (0.031)
MS	0.288*** (0.024)	0.258*** (0.024)	0.784*** (0.073)	0.514*** (0.143)
HS	−0.035** (0.014)	0.056*** (0.015)	−0.304*** (0.043)	0.049 (0.095)
PI	0.036** (0.018)	0.052*** (0.019)	0.133*** (0.051)	0.431*** (0.113)
BB	0.191*** (0.021)	0.150*** (0.022)	0.339*** (0.058)	0.008 (0.130)
NH	0.087*** (0.015)	0.070*** (0.016)	0.200*** (0.040)	0.348*** (0.083)
FA	0.148*** (0.007)	0.159*** (0.006)	0.144*** (0.016)	0.590*** (0.034)
FI	0.102*** (0.007)	0.116*** (0.007)	0.159*** (0.015)	0.351*** (0.035)
Constant	7.659*** (0.144)	7.208*** (0.153)	6.789*** (0.407)	−5.935*** (0.858)
*N*	8,009	8,008	8,009	7,797
*F*-value	337.066***	424.374***	119.167***	341.029***
*R* ^2^	0.422	0.465	0.189	0.294

***, **, and * indicate significance at the 1%, 5%, and 10% confidence levels, respectively, with standard errors in brackets and coefficients outside brackets.

### Education level heterogeneity

Table 9 reports the model regression results after introducing the interaction term (*Risk*×*EL*) for risk attitudes and education level. When the risk attitude is affected by the education level, the regression coefficients of the interaction terms in all kinds of consumption are not significant, which statistically proves that the interaction effect between the risk attitude and the education level does not exist. Therefore, difference exists in the impact of risk attitude on household consumption behavior based on education level, and no linkage effect exists between risk attitude and education level.

**Table 9:** Results of education level heterogeneity analysis.

	**(1)**	**(2)**	**(3)**	**(4)**
	**Total consumption**	**Subsistence consumption**	**Developmental consumption**	**Enjoyment consumption**
Risk	0.064** (0.027)	0.092*** (0.028)	0.203*** (0.074)	0.467*** (0.160)
Risk × EL	0.000 (0.006)	−0.006 (0.006)	−0.024 (0.015)	−0.008 (0.031)
GE	−0.072*** (0.017)	−0.079*** (0.018)	−0.200*** (0.050)	−0.572*** (0.107)
AG	−0.016*** (0.003)	−0.005 (0.003)	−0.013 (0.010)	−0.091*** (0.020)
AG2	0.000** (0.000)	−0.000*** (0.000)	−0.000 (0.000)	0.001*** (0.000)
EL	0.055*** (0.010)	0.065*** (0.010)	0.130*** (0.028)	0.606*** (0.058)
MS	0.288*** (0.024)	0.256*** (0.024)	0.785*** (0.073)	0.512*** (0.143)
HS	−0.035** (0.014)	0.055^***^ (0.015)	−0.304*** (0.043)	0.048 (0.095)
PI	0.036** (0.018)	0.051*** (0.019)	0.131*** (0.051)	0.430*** (0.113)
BB	0.191*** (0.021)	0.149*** (0.022)	0.338*** (0.058)	0.007 (0.130)
NH	0.088*** (0.015)	0.071*** (0.016)	0.201*** (0.040)	0.350*** (0.083)
FA	0.148*** (0.007)	0.159*** (0.006)	0.144*** (0.016)	0.590*** (0.034)
FI	0.102*** (0.007)	0.116*** (0.007)	0.159*** (0.015)	0.351*** (0.035)
Constant	7.766*** (0.121)	7.287*** (0.125)	6.220*** (0.337)	−5.765*** (0.697)
*N*	8,009	8,008	8,009	7,797
*F*-value	337.815***	424.639***	119.096***	346.136***
*R* ^2^	0.422	0.465	0.189	0.294

*** and ** indicate significance at the 1% and 5% confidence levels, respectively, with standard errors in brackets and coefficients outside brackets.

## Robustness test

Considering the possible endogeneity problems in this paper, which may lead to the bias of the main results. To ensure the robustness and credibility of the research findings, this article sets the analysis object as the age-appropriate consumer group with more household consumption behaviors, and the dominant consumer group in the age range of 22–37 years old. On this basis, the OLS model of the impact of risk attitude on household consumption behavior was reconstructed to test the robustness.

### Impact of risk attitude on household total consumption behavior

Table 10 demonstrates that the robustness test results are basically consistent with the model results in Table 3, and the relevant result coefficients are higher compared with the previous ones, which shows that the risk attitude significantly impacts residents' household consumption behavior. Amongst the leading consumer groups aged 22–37, it can be found that risk preference and risk neutrality always positively impact household consumption behavior, and risk aversion always negatively impacts household consumption behavior, which is more significant than the previous regression results. In addition, amongst the three risk attitudes, the positive impact of risk preference on household consumption is more significant. Conclusively, the robustness analysis with the help of the method of controlling samples shows that the significant impact of risk attitude on household consumption behavior is robust, which indicates the reliability of our findings.

**Table 10:** The impact of risk attitude on household total consumption behavior.

	**(1)**	**(2)**	**(3)**	**(4)**	**(5)**
	**Total consumption**	**Total consumption**	**Total consumption**	**Total consumption**	**Total consumption**
Risk	0.252*** (0.010)	0.092*** (0.011)			
Risk-P			0.128*** (0.023)		
Risk-N				0.116*** (0.016)	
Risk-A					−0.051*** (0.013)
GE		−0.065*** (0.017)	−0.076*** (0.016)	−0.073*** (0.016)	−0.075*** (0.016)
AG		0.019*** (0.005)	0.025*** (0.004)	0.024*** (0.005)	0.025*** (0.005)
AG2		−0.000*** (0.000)	−0.001*** (0.000)	−0.001*** (0.000)	−0.001*** (0.000)
EL		0.071*** (0.005)	0.083*** (0.005)	0.082*** (0.005)	0.085*** (0.005)
MS		0.299*** (0.024)	0.309*** (0.021)	0.304*** (0.022)	0.309*** (0.022)
HS		−0.006 (0.015)	−0.012 (0.013)	−0.012 (0.013)	−0.011 (0.013)
PI		−0.014 (0.017)	−0.009 (0.017)	−0.008 (0.017)	−0.009 (0.017)
BB		0.243*** (0.021)	0.268*** (0.020)	0.266*** (0.020)	0.270*** (0.020)
NH		0.094*** (0.016)	0.099*** (0.015)	0.101*** (0.015)	0.100*** (0.015)
FA		0.143*** (0.007)	0.142*** (0.005)	0.141*** (0.005)	0.143*** (0.005)
FI		0.103*** (0.007)	0.096*** (0.006)	0.096*** (0.006)	0.097*** (0.006)
Constant	10.472*** (0.018)	7.019*** (0.082)	7.148*** (0.067)	7.150*** (0.067)	7.148*** (0.067)
*N*	8,335	5,663	7,610	7,610	7,608
*F*-value	575.673***	350.258***	484.557***	490.187***	482.647***
*R* ^2^	0.051	0.409	0.423	0.424	0.422

***indicates significance at the 1% confidence level, with standard errors in brackets and coefficients outside brackets.

### Impact of risk preference on household total consumption behavior

Table 11 reports the robustness test results of the impact of risk preference on different consumption behaviors of residents' families. The results are basically consistent with the results in Table 4, and the relevant result coefficients are higher than the previous ones, which shows that risk preference significantly impacts residents' consumption behaviors. The risk preference of the dominant consumer groups aged 22–37 has always had a positive impact on household survival consumption, development consumption and enjoyment consumption, and it is more significant than the previous regression results. Moreover, the impact of risk preference on household consumption for enjoyment is still greater than the impact on household consumption for survival and development.

**Table 11:** The impact of risk preference on household total consumption behavior.

	**(1)**	**(2)**	**(3)**
	**Subsistence consumption**	**Developmental consumption**	**Enjoyment consumption**
Risk-P	0.162*** (0.024)	0.314*** (0.061)	0.787*** (0.131)
GE	−0.069*** (0.017)	−0.131*** (0.047)	−0.562*** (0.098)
AG	0.034*** (0.005)	−0.066*** (0.013)	0.062** (0.026)
AG2	−0.001*** (0.000)	−0.002*** (0.000)	−0.001 (0.001)
EL	0.090*** (0.005)	0.170*** (0.013)	0.711*** (0.028)
MS	0.323*** (0.022)	0.793*** (0.064)	0.488*** (0.122)
HS	0.100*** (0.014)	−0.237*** (0.039)	0.135 (0.085)
PI	−0.037** (0.017)	−0.069 (0.047)	0.298*** (0.103)
BB	0.263*** (0.021)	0.577*** (0.054)	0.368*** (0.120)
NH	0.091*** (0.015)	0.233*** (0.039)	0.392*** (0.081)
FA	0.152*** (0.005)	0.141*** (0.014)	0.539*** (0.028)
FI	0.113*** (0.006)	0.156*** (0.013)	0.335*** (0.029)
Constant	6.691*** (0.069)	4.943*** (0.183)	−8.247*** (0.339)
*N*	7,609	7,610	7,335
*F*-value	576.368***	160.006***	489.523***
*R* ^2^	0.457	0.179	0.305

*** and ** indicate significance at the 1% and 5% confidence levels, respectively, with standard errors in brackets and coefficients outside brackets.

### Impact of risk neutrality on household total consumption behavior

The regression results in Table 12 are basically consistent with the model results in Table 5, and the coefficients of the correlation results are higher than those of the previous results, which shows that risk neutrality significantly impacts household consumption behavior. Similar to the full sample, in the test results of sub-samples, risk neutrality always positively impacts household consumption for survival, development, and enjoyment, and it is more significant than the previous regression results. Moreover, the impact of risk neutrality on household enjoyment consumption remains greater than that on household survival consumption and development consumption.

**Table 12:** The impact of risk neutrality on household total consumption behavior.

	**(1)**	**(2)**	**(3)**
	**Subsistence consumption**	**Developmental consumption**	**Enjoyment consumption**
Risk-N	0.134*** (0.017)	0.296*** (0.048)	0.762*** (0.101)
GE	−0.065*** (0.017)	−0.123*** (0.047)	−0.547*** (0.098)
AG	0.033*** (0.005)	−0.068*** (0.013)	0.054** (0.026)
AG2	−0.001*** (0.000)	0.002*** (0.000)	−0.001 (0.001)
EL	0.089*** (0.005)	0.168*** (0.013)	0.706*** (0.028)
MS	0.318*** (0.022)	0.782*** (0.064)	0.464*** (0.122)
HS	0.100*** (0.014)	−0.239*** (0.039)	0.130 (0.085)
PI	−0.036** (0.017)	−0.068 (0.047)	0.302*** (0.103)
BB	0.262*** (0.021)	0.572*** (0.054)	0.354*** (0.120)
NH	0.094*** (0.015)	0.238*** (0.038)	0.402*** (0.080)
FA	0.152*** (0.005)	0.141*** (0.014)	0.538*** (0.028)
FI	0.112*** (0.006)	0.155*** (0.013)	0.331*** (0.029)
Constant	6.691*** (0.069)	4.950*** (0.183)	−8.221*** (0.339)
*N*	7,609	7,610	7,335
*F*-value	582.540***	161.171***	497.829***
*R* ^2^	0.457	0.180	0.307

*** and ** indicate significance at the 1%, and 5% confidence levels, respectively, with standard errors in brackets and coefficients outside brackets.

### Impact of risk aversion on household total consumption behavior

The robustness test results of risk aversion are shown in Table 13. From Table 13, we can see that the pair regression coefficients of risk aversion are all negative and significant, which indicates that risk aversion has a significant negative impact on household consumption behavior. Different from the results in Table 6, in the dominant consumer group aged 22–37, risk aversion has a significant inhibitory effect on survival consumption, development consumption, and enjoyment consumption behavior, whereas the inhibitory effect in the whole sample is not significant. A possible reason is that risk aversion only affects certain groups and has no significant effect on other age groups. The corresponding coefficients of survival consumption, development consumption and enjoyment consumption are −0.028, −0.099, and −0.143, respectively, indicating that the inhibitory effect of risk aversion on enjoyment consumption is greater than that on survival consumption and development consumption.

**Table 13:** The impact of risk aversion on household total consumption behavior.

	**(1)**	**(2)**	**(3)**
	**Subsistence consumption**	**Developmental consumption**	**Enjoyment consumption**
Risk-N	−0.028** (0.014)	−0.099*** (0.038)	−0.143* (0.082)
GE	−0.066*** (0.017)	−0.126*** (0.047)	−0.552*** (0.098)
AG	0.035*** (0.005)	−0.065*** (0.013)	0.067** (0.026)
AG2	−0.001*** (0.000)	0.002*** (0.000)	−0.001 (0.001)
EL	0.092*** (0.005)	0.174*** (0.013)	0.723*** (0.028)
MS	0.321*** (0.022)	0.792*** (0.064)	0.484*** (0.122)
HS	0.102*** (0.014)	−0.234*** (0.039)	0.142* (0.086)
PI	−0.038** (0.018)	−0.072 (0.048)	0.288*** (0.103)
BB	0.267*** (0.021)	0.582*** (0.054)	0.384*** (0.120)
NH	0.094*** (0.015)	0.236*** (0.039)	0.403*** (0.081)
FA	0.154*** (0.005)	0.145*** (0.014)	0.547*** (0.028)
FI	0.114*** (0.006)	0.158*** (0.013)	0.339*** (0.029)
Constant	6.679*** (0.069)	4.934*** (0.183)	−8.307*** (0.341)
*N*	9,953	9,954	9,680
*F*-value	571.387***	157.229***	485.148***
*R* ^2^	0.454	0.177	0.303

^***^, ^**^, and ^*^ indicate significance at the 1%, 5%, and 10% confidence levels, respectively, with standard errors in brackets and coefficients outside brackets.

## Conclusions and implications

### Conclusions

On the basis of the micro data of the 2017 CHFS. We constructed OLS models to empirically test the impact of risk attitude on household consumption behavior. The study found that the risk attitude of household heads will have a significant positive impact on their family's consumption behavior, in which the risk preference positively impacts the consumption behavior of resident families. When the risk attitude of residents is more inclined to risk preference, it can promote more developmental consumption and enjoyment consumption, and risk aversion will negatively impact the consumption behavior of specific groups. In the heterogeneity analysis, we conclude that the impact of risk attitude on different gender and age is also quite different. The results show that for male households, risk attitude remains an important factor in consumption behavior. When men's risk attitude tends toward risk aversion, it can promote more survival consumption, whereas women's risk attitude has no effect on household consumption behavior. Moreover, with the increase of age, risk attitude remains an important factor in consumer behavior. When residents' risk attitude tends toward risk preference, it can promote more developmental consumption. In addition, our main research conclusions passed the robustness test, indicating that risk attitude is an important factor affecting household consumption behavior.

### Implications

Referring to the above empirical findings, risk attitude is a vital factor affecting residents' household consumption behavior; therefore, we can promote consumption by enhancing residents' risk attitude. To better improve residents' risk attitude, better promote residents' consumption, achieve sustained, high-quality, and rapid economic development and alleviate the pressure of national employment, this paper puts forward the following policy suggestions.

Firstly, the construction of consumption environment should be strengthened. Consumers' consumption environment will affect risk aversion, and residents facing income uncertainty will show a higher degree of absolute risk aversion. For most consumers, meeting their needs is the most practical. In the above demonstration, we found that risk attitude will affect the occurrence of consumption behavior. In the face of perfect consumer market system and action mechanism, residents will be willing to take appropriate risks for consumption to meet their needs. When they cannot see the state of the consumer market, they frequently opt for consumer goods with high brand awareness to keep the risk within a manageable range. To encourage residents to actively participate in consumption, the government should strengthen the construction of consumption environment, create a safe and stable consumption environment, and establish a perfect consumer market supervision system and risk control system, so that residents can accept appropriate risks to choose consumer goods.

Secondly, residents' sense of security and happiness should be improved. Safety and happiness factors will positively impact risk attitude. Therefore, residents' consumption can be promoted by improving residents' sense of security and happiness. Presently, residents' sense of security mostly comes from public security. To improve residents' sense of security, the basic safety of residents must be ensured by eradicating theft, robbery, and vandalism. The happiness of residents comes from the living environment of residents, such as community hygiene and some convenience measures. To improve the well-being of community residents, residents should be allowed to enjoy the convenience of service facilities. For the community, basic service facilities mainly include education, medical care, basic services, catering, and entertainment. A reasonable layout of service facilities can well-serve the daily life of residents, and create a more convenient living environment, thereby enhancing residents' happiness.

Thirdly, the reform of the income distribution system should be expedited. The risk preference of households increases with the increase of income level. Thus, household consumption can be stimulated by promoting the reform of the income distribution system. Presently, the income of urban and rural residents is quite different, which is not conducive to the common prosperity of all residents. The income distribution system can reflect the social fairness of the country to a certain extent. Therefore, to better promote residents' consumption, boost national economic development, and enhance social harmony, a reasonable income distribution system must be formulated, and the construction of various social welfare and social security systems must be strengthened for residents.

Fourthly, the residents' financial literacy should be improved. Financial literacy significantly impacts residents' risk attitude. When residents have a high level of financial literacy, they will enhance their sense of grasp of financial activities and show a higher risk preference. Therefore, residents' consumption can be promoted by improving residents' level of financial literacy (66). To improve the financial literacy level of residents, inclusive financial literacy education must be promoted. Financial institutions can introduce relevant financial knowledge to residents by conducting financial publicity activities. Financial institutions can also broaden access to financial knowledge and conduct more comprehensive publicity to residents through the Internet or news media.

Fifthly, the financial market order must be further improved. The improvement of financial market order is conducive to enhancing residents' awareness of risks and increasing residents' risk preference. Presently, China's financial market still has problems, such as imperfect supervision, which causes residents to face many uncertainties when making decisions and reduces their risk preference. Therefore, policy makers should continue to vigorously maintain the order of the financial market, promote the healthy development of the financial market, and increase the level of residents' risk preference by reducing the uncertainty of residents' economic decision making. Diversifying the risk attitudes of residents can not only optimize the consumption structure of residents, but also promote the optimization of the consumption structure of residents.

Finally, education on risk knowledge should be strengthened to improve residents' mental health. The diversification of risk attitudes is an important factor to reflect the health of residents. Therefore, the government should publicize risk knowledge through media advertising and training to boost residents' risk preference and promote the development of residents' risk diversification, thereby improving public health.

### Research limitations and future research prospects

Although this paper uses the 2017 CHFS data to thoroughly analyse the impact of risk attitudes on residents' consumption, examining the changes in residents' risk attitudes on consumption after the COVID-19 pandemic was difficult due to data limitations. If the latest data are available, the impact of Chinese residents' risk attitudes on consumption can be further examined. This paper provides a research perspective for analyzing the impact of residents' mental health on individual behavioral decisions. Future research can also analyse the impact of residents' mental health on other individual economic behaviors, such as individual investment choices, employment choices, and so on. In addition to individual economic behavior, how an individual's social behavior is affected by mental health can be studied. Future research can analyse why residents' mental health affects individual behavior from the perspectives of society, economy, culture, and psychology.

## Data availability statement

The raw data supporting the conclusions of this article will be made available by the authors, without undue reservation.

## Author contributions

ZT: conceptualization and supervision. XX: writing-original draft and software. SX and ZT: writing-review and editing. XX and SX: methodology and data curation. All authors contributed to the article and approved the submitted version.

## Funding

This research is funded by the Philosophy and Social Science Planning Project of Guangdong Province (GD21CYJ31), Characteristic Innovation Project of Ordinary Colleges and Universities Guangdong Province (2021WTSCX206), Guangzhou Philosophy and Social Science Planning Project in 2022 (2022GZYB12), and the Fundamental Research Funds for Central Universities in China (202210403).

## Conflict of interest

The authors declare that the research was conducted in the absence of any commercial or financial relationships that could be construed as a potential conflict of interest.

## Publisher's note

All claims expressed in this article are solely those of the authors and do not necessarily represent those of their affiliated organizations, or those of the publisher, the editors and the reviewers. Any product that may be evaluated in this article, or claim that may be made by its manufacturer, is not guaranteed or endorsed by the publisher.
